# The Effects of β _2_ -Adrenoreceptor Activation on the Contractility, Ca-Signals and Nitric Oxide Production in the Mouse Atria

**Published:** 2011

**Authors:** Yu.G. Odnoshivkina, A.M. Petrov, A.L. Zefirov

**Affiliations:** Kazan State Medical University, Federal Agency for Health Care and Social Development

**Keywords:** β_2_-adrenoreceptor, fenoterol, calcium, nitrico xide, contractility, atrial cardiomyocytes

## Abstract

The effects of the selective β_2_-adrenoreceptor agonist (fenoterol) on the functioning of mouse atrial were studied using both tensometry and fluorescent methods. It has been demonstrated that with the use of a high concentration of fenoterol (in the range of 1–50 µM), there is a more significant positive inotropic effect observed within a shorter period of time. In the case of relatively low doses of fenoterol (1 and 5 µM), its contractility effects are observed 20 min after the application of agonist, whereby in the case of high concentrations (25, 50 and 300 µM), the effects appear within the first minutes. During the first 10–15 min, 5 µM fenoterol causes an increase in the amplitude of Ca-signals in cardiomyocytes (this indicates an increase in the concentration of Ca ions during systole) and the activation of NO synthesis. However, after 20 min, the production of NO decreases; while the amplitude of Ca-signals remains high. The application of 50 µM fenoterol leads to a rapid increase in the amplitude of Ca-signals: at the same time, it causes a decrease in the production of NO, which we found to begin to increase after 10 min of agonist application. It is suggested that the dynamics of the positive inotropic effect occurring under pharmacological stimulation of β_2_-adrenoreceptors depend on the rate of increase in the amplitude of Ca-signals and on the degree of NO synthesis.

## INTRODUCTION 

One of the mechanisms that regulate the action of the heart is based on the interaction of catecholamines (adrenaline and noradrenaline) with G protein-coupled β-adrenoreceptors in cardiomyocytes [1, 2]. Depending on their sensitivity to pharmacological agents and on their structural features, β-adrenoreceptors are divided into three types: β _1_ , β _2_ , and β _3_ . All types of β-adrenoreceptors are expressed within the heart. The function of the β _1_ -adrenoreceptors in the heart is well known. Meanwhile, β _2_ -adrenoreceptors have been subjected to much less thorough study: only their functions in the regulation of the vascular tone in the heart and the skeletal muscles of bronchial lumen and the metabolism of a body have [3].

The concentration of β _2_ -adrenoreceptors is particularly high in atrial cardiomyocytes. While, within the entire heart, the content of β _2_ -adrenoreceptors reaches 30–40% of the total amount of β-adrenoreceptors [2], in human atrial cardiomyocytes their content is over 55% [4]. The stimulation of β _2_ -adrenoreceptors leads to an increase in the contractility of the ventricular cardiomyocytes in a rat heart, with no significant impact on the rate of relaxation [5]. It is believed that the activation of β _2_ -adrenoreceptors causes a positive inotropic reaction without increasing the risk of arrhythmias and sudden death of the animal [6]. It should be noted that, upon stimulation of β _2_ -adrenoreceptors, the inotropic effect might be negative, e.g. in papillary muscles of the guinea pig heart [7].

The effect of the stimulation of β _2_ -adrenoreceptors on the contractility of ventricular cardiomyocytes is defined by the interaction of the receptor with the Gs protein, which initiates the adenylyl cyclase cascade causing the activation of protein kinase A. Under the action of this kinase, part of the L-type calcium channels could be opened and the conduction of these channels enhanced, and consequently the amount of Ca ions entering the cytoplasm during action potential could also increase [8, 9]. However, the activated β _2_ -adrenoreceptors stimulate also Gi proteins, which inhibit the adenylyl cyclase cascade, thereby limiting the positive inotropic effect produced by the β _2_ -adrenoreceptor agonists [10, 11]. The activation of Gi proteins initiates the phosphoinositol-3-kinase (PI3K)/protein kinase B cascade, which is aimed at maintaining cell survival, protecting them from the action of reactive oxygen speciesand hypoxia [12, 13]. In addition, the phosphoinositol-3-kinase (PI3K)/protein kinase B cascade may initiate an increase in the production of nitric oxide (NO), which, by affecting the guanylate cyclase system, is capable of inhibiting the effect of β _2_ -adrenoceptor agonists [14, 15] and preventing the desensitization of β _2_ -adrenoceptors [16] as well. It has been suggested that β _2_ -adrenoreceptors play a cardioprotective role, while β _2_ -adrenoreceptor agonists can be viewed as promising pharmacological agents [13]. Thus, in mice with overexpression of β _2_ -adrenoreceptors, no heart failure develops and no signs of cardiomyocyte damage are revealed against a background of increased frequency and strength of heart contractions, which are observed even in the absence of β _2_ -adrenoreceptor activation by endogenous ligands [17]. The point is that β _2_ -adrenoreceptors can be spontaneously switched into their active state regardless of whether there are agonists in the extracellular medium or not [18]. It is interesting that, under spontaneous activation of β _2_ -adrenoreceptors, contractility increases, but the entering calcium current through L-type calcium channels does not change [19].

β _2_ -Adrenoreceptors are very “flexible” molecules with several active states (conformations). The conformations define the properties of the receptors, including their ability to bind to effector signaling proteins, whose role can be played not only by G proteins, but also by tyrosine kinase Src, by the factor that controls the Na-H exchange, by arrestin, by the N-ethylmaleimide factor, and by several scaffold proteins [20–24]. It was demonstrated recently that different agonists can transform β _2_ -adrenoreceptors into forms with specific active conformations; hence the reason why the type of agonist defines the cell-mediated response (this phenomenon is called the “functional selectivity” of agonists) [25]. Fenoterol is one of the most widely used drugs in clinical medicine and one of the effective short-acting β _2_ -adrenoreceptor agonists. Its effects include a significant increase in the cAMP level and the activation of mitogen-activated protein (MAP) kinase [25] in human airway smooth muscle cells and, in endotheliocytes, intensification of the synthesis of nitric oxide (NO) [26]. It should be noted that, under the action of fenoterol, β _2_ -adrenoreceptors undergoes intensive phosphorylation by G protein-coupled receptor kinases, followed by their internalization during clathrin-mediated endocytosis. As a result, long-term use of fenoterol leads to desensitization and a decrease in the amount of the β _2_ -adrenoreceptors on the surface of bronchial epithelium [25, 27]. New data on the unique properties of fenoterol and its stereoisomers has appeared; this data can be used to design drugs that have a high selectivity value and pronounced cardioprotective properties [28, 29].

The role of β _2_ -adrenoreceptors in atrial cardiomyocytes is not thoroughly understood. There is data indicating that there is an increase in contractility and the rate of intake of the calcium current upon stimulation of the atrial β _2_ -adrenoreceptors in guinea pigs, cats, and humans [15, 30, 31]. However, in mice with overexpression of human β _2_ -adrenoreceptors, the activation of atrial β _2_ -adrenoreceptors by isoprenaline has a negative inotropic effect, while, under normal circumstances, isoprenaline, which affects mostly β _1_ -adrenoreceptors, has a positive inotropic effect [32, 33]. In this work, we studied how various doses of racemic fenoterol, a β _2_ -adrenoreceptor agonist widely used in medicine, influence the atrial contractility, amplitude of Ca-signals, and NO production.

## EXPERIMENTAL 

Isolated atria of white mice were used in the experiments. The standard Krebs solution for warm-blooded animals with the following composition was used: 144.0 mM NaCl, 5.0 mM KCl, 0.1 mM MgCl _2_ , 2.0 mM CaCl _2_ , 1.0 mM NaH _2_ PO _4_ , 2.4 mM NaHCO _3_ , 11.0 mM glucose; the solution was saturated with oxygen. The pH value of the solutions was maintained at 7.2–7.4 at a temperature of 20°C. During the experiment, the specimen was stimulated by electrical pulses of suprathreshold amplitude at a frequency of 0.1–1 Hz via platinum electrodes. In the majority of experiments, the application (20 min) of (±)-fenoterol (Sigma, USA), a β _2_ -adrenoreceptor agonist, at concentrations of 1–300 µM was used. In some cases, 0.1 µM ICI-118.551 (Tocris, USA), a selective blocker of β _2_ -adrenoreceptors was applied.

**Tensometry**

The atrial contraction was registered using a PowerLab installation. One end of the isolated atrium was tied to a fixed nail, and the other end was linked to a nail connected to a strain gauge with a sensitivity of 0–25 g (AD Instruments). The signals were treated using Chart software; the contractility was determined in grams. 

**Fluorescent Microscopy**

The fluorescent experiments were carried out using an OLYMPUS CX41 (with exchangeable monochromatic excitation light sources) and an OLYMPUS BX51 (equipped with the DSU confocal system) microscope with a LMPlanFI 20×/0.40 and UPlanSApo 60×/1.20W objectives. The images were made using high-speed CCD cameras produced by OLYMPUS: a F-View II black and white and DP71 color CCD camera. The images were treated using Cell^A, Cell^P, and ImagePro software. The fluorescence intensity was estimated in relative units (rel. units) that correspond to the brightness value in pixels. 

*Measurements of the Intracellular Concentration of Calcium Ions*. The changes in the concentration of Ca ^2+^ were determined using a Fluo-4 dye, which allows the Ca ^2+^ concentration to be precisely measured in a range from 1 µM to 1 mM. Fluo-4 is weakly fluorescent in the absence of Ca ^2+^ , but its binding to Ca ions leads to an increase in the fluorescence value by a factor over 100 [34]. Fluo-4-AM (Molecular Probes, USA), a membrane-penetrating form of the dye, was used; it was dissolved in DMSO (dimethyl sulfoxide, Sigma, USA) and stored in the frozen form (for not more than a week) in the dark. Just prior to the experiment, a Pluronic F-127 (Molecular Probes, USA) nonionic detergent was added into a Fluo-4-AM batch; the detergent facilitates the dissolving of nonpolar Fluo-4-AM in an aqueous (physiological) solution. In a work solution, the final concentration of Fluo-4-AM was 1 µM, and the content of DMSO and Pluronic F-127 was not more than 0.0005%. The isolated atrial specimen was held in the solution containing 1 µM Fluo-4-AM for 20 min at room temperature. Afterwards, the specimen was perfused with a physiological solution for 40 min (during this period of time, deesterification of Fluo-4-AM in the cytoplasm ended and the formation of hydrophilic Fluo-4, which cannot pass through the membrane into the intracellular medium [34], occurred). Thereafter, measurements of the fluorescence in the cardiomyocytes of the isolated atria were made. The fluorescence of the dye was excited by short (about 1 s) flashes of light with a wavelength of 480 nm and registered using an emission-color filter that transmits light with a wavelength of above 515 nm. During the contraction-relaxation cycle of the atrial specimen, periodic changes in the fluorescence of the Ca sensor, which appeared in the form of flashes (“Ca-signals”), were observed: the intensity of the fluorescence increased during contraction and decreased during relaxation. Ca-signals indicate an increase in the concentration of Ca ions, which initiate contraction of cardiomyocytes. The minimum fluorescence value observed during diastole was subtracted from the maximum fluorescence value observed during systole, in order to estimate the amplitude of the Ca-signals.

*Measurements of the Concentration of Nitric Oxide (NO).*The production of nitric oxide was detected using a DAF-FM-diacetate marker (Molecular Probes, USA), which was excited by light with a wavelength of λ = 495 nm; for the registration of fluorescence, an emission filter that transmits light with a wavelength above 515 nm was used. DAF-FM-diacetate easily permeates through the cellular membranes. Inside the cell, DAF-FM-diacetate is deacetylated by intracellular esterase to DAF-FM. Prior to reacting with NO, DAF-FM almost does not fluoresce; but its interaction with NO leads to an increase in the fluorescence intensity by a factor of more than 160 [29]. DAF-FM was dissolved in DMSO and stored in frozen form in a dark place. The specimen of the isolated atrium was held in a solution containing 2 µM DAF-FM-diacetate for 30 min at room temperature. After that, the atrial specimen was perfused with a physiological dye-free solution for 20 min (the period of time needed for deacetylation of the marker to finish [35]). Thereafter, the measurements of fluorescence in cardiomyocytes of the isolated atria were made.

A statistical analysis was performed using the Origin Pro software. The results of the measurements were presented as mean values ± standard error ( *n * is the number of independent trials). The significance of the differences was determined in accordance with the Student’s test and ANOVA.

## RESULTS 

**Dose-Dependence of the Inotropic Effect of Fenoterol**

**Fig. 1:**
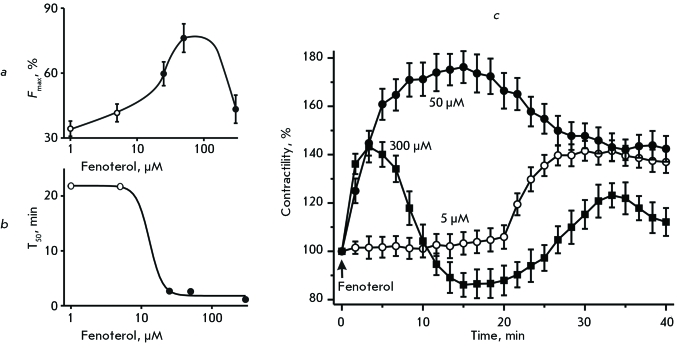
Effect of fenoterol on the contractility of the isolated atria. The influence of the various doses of fenoterol on contractility ( *a* ) and time-course of the effect ( *b* ).Y-axis: *а* is the maximum amplitude of contractions (F _max_ , “0” corresponds to the value observed prior to application of fenoterol ); *b * is the period of time from the moment fenoterol was applied to the moment when the amplitude reached 50% of the maximum amplitude of contractions (T _50_ ). X-axis: the logarithmic scale of concentrations (µM). ( *c* ) The change in the atrial contractility under the action of 5, 50, and 300 µM of fenoterol (open and filled circles and filled squares, respectively). Y-axis: the force of contraction, (%) (100% corresponds to the level of contractility prior to the application of fenoterol). The beginning of the application of fenoterol is indicated by an arrow. The presented data on the change in the atrial contractility under the action of fenoterol applied at concentrations of 1 ( *n * = 5), 5 ( *n * = 5), 25 ( *n * = 6), 50 ( *n*  = 8), 300 ( *n*  = 5) µM were obtained in independent experiments.

*Amplitude of Contractions. *The addition of fenoterol at concentrations ranging from 1 to 300 µM led to a significant increase in contractility ( *Fig. 1a, b* ). The application of 1 and 5 µM of fenoterol increased contractility to 134 ± 4.4% ( *p*  < 0.01, *n*  = 5) and 144.6 ± 5.1% ( *p*  < 0.01, *n*  = 5), relative to the control value, respectively. Under the action of fenoterol (25 and 50 µM), contractility increased even more; i.e. up to 159.7 ± 5.5% ( *p*  < 0.01, *n*  = 6) and 176.2 ± 6.6% ( *p*  < 0.01, *n*  = 8), respectively. However, 300 µM of fenoterol caused an increase in contractility only to 143.3 ± 6.5% ( *p*  < 0.01, *n*  = 5) ( *Fig. 1c* ). Since β _2_ -adrenoreceptors are desensitized relatively fast by high doses of agonists, the slight influence of 300 µM of fenoterol on contractility most likely relates to desensitization processes [27, 36].

*Time-Course of the Effect. *The rate of development of the positive inotropic effect of fenoterol varied depending on its concentration: the higher the concentration, the earlier the increase in contractility was observed ( *Fig. 1a, b* ). The amplitude began to grow only 20 min after fenoterol at concentrations of 1 and 5 µM was applied, and maximum contractility was observed after 30–40 min. When fenoterol was used at concentrations of 25 and 50 µM, the contractility increased significantly faster: i.e., 15 and 13 min after the addition of the agonist, contractility reached a maximum. 300 µM of fenoterol led to maximum contractility just after 3 min.

*Delayed Effect of Fenoterol. *As was mentioned above, at low concentrations of fenoterol, the positive inotropic effect occurs very late, only after 20–25 min ( *Fig. 1c* ). In order to clarify why the development of the effect is so slow, additional experiments in which a solution of fenoterol (5 µM) was replaced by a normal Krebs solution after 20 min (i.e. prior to the beginning of the increase in contractility). The contractility was found to increase despite the absence of fenoterol in the solution. Ten minutes after the removal of fenoterol from the solution surrounding the atrial specimen, the amplitude reached its maximum: 141.6 ± 4.1% relative to the control value ( *p*  < 0.01, *n*  = 8). It then decreased gradually. This indicates that the delayed effect observed at low concentrations of β _2_ -adrenoreceptors is related to the activation of intracellular signal systems, whose action develops at a very low rate.

In further experiments, only two concentrations of fenoterol were used: 5 µM, at which the delayed effect was observed; and 50 µM, at which contractility was found to increase rapidly ( *Fig. 1c* ). It should be noted that, in the presence of the selective β _2_ -adrenoreceptor blocker (0.1 µM ICI-118.551), no positive inotropic effect of fenoterol at concentrations of 5 and 50 µM appears (data are not presented).

**The Effect of Fenoterol on Ca-Signals**

**Fig. 2:**
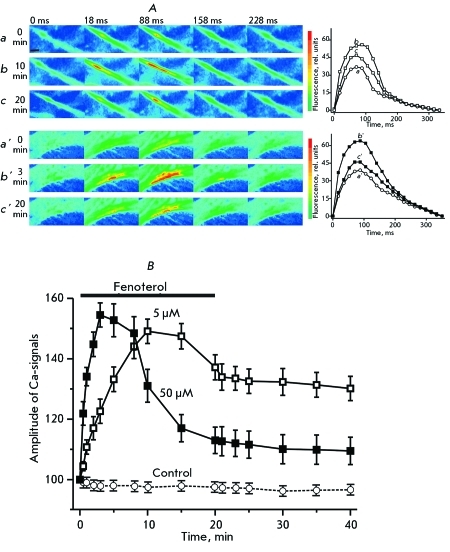
Effect of fenoterol on the Ca-signals in cardiomyocytes of the isolated atria. )The changes in fluorescence of the Fluo-4 Ca-indicator loaded into cardiomyocytes of the isolated atrium. The pseudo-colored images illustrate Ca-signals occurring in cardiomyocytes of the isolated atria per single atrial contraction before ( *a* , *a* ’) and after the application of fenoterol at concentrations of 5 ( *b, c* ) and 50 µM ( *b’, c’* ). Above the images, the time from the stimulus is shown (ms): 0, 18, 88, 158, 228. The scale is 10 µm. On the right, the curves representing changes in the fluorescence of Fluo-4 occurring as a response to the stimulus are shown. Along the Y-axis: the intensity of fluorescence, rel. units (“0” is fluorescence before stimulus); along the X-axis: time in ms from the stimulus. ( *B)* The effect of 5 ( *n*  = 7) and 50 ( *n*  = 7) µM fenoterol on the amplitude of Ca-signals (open and filled squares, respectively). Changes in the amplitude of Ca-signals in the absence of fenoterol are shown with open circles connected by a dash line (Control, *n*  = 5). Along the Y-axis: fluorescence (%); along the X-axis: time (min). The application of fenoterol is indicated with a line.

The dynamics of the intracellular concentration of calcium ions, which initiate the contractions of cadriomyocytes, significantly change under the action of fenoterol ( *Fig. 2* ). Use of a low concentration of fenoterol (5 µM) leads to a gradual increase in the amplitude of Ca-signals. After 3 min of application of the β _2_ -adrenoreceptor agonist, the amplitude of Ca-signals reached 122.6 ± 4.7% ( *p*  < 0.05, *n*  = 7); and after 10 min, the value of the amplitude was 152.1 ± 4.9% ( *p*  < 0.01, *n*  = 7) relative to the initial values. After 15 min of application of the agonist, the amplitude of Ca-signals somewhat declined, but after 20 min, it stabilized at a level of 130–140%. Five minutes after the surrounding solution was replaced with a fenoterol-free solution, the amplitude of Ca-signals was 133.6 ± 4.7% ( *p*  < 0.01, *n*  = 7). The amplitude of Ca-signals returned to the basal level 60–70 min after fenoterol was removed from the intracellular solution. The application of fenoterol at high concentrations (50 µM) caused an increase in the amplitude of Ca-signals to 121.9 ± 4.9% ( *p*  < 0.05, *n*  = 7) just after 30 s, and by the third minute, its value reached a maximum, 154 ± 4.8% ( *p*  < 0.01, *n*  = 7). After 8 min of application, the amplitude began to decline, and 20 min after the application of fenoterol, its value was 111.2 ± 4.3% ( *p*  < 0.05, *n*  = 7). The amplitude of Ca-signals was brought to its initial value 50–60 min after perfusion of the atrial specimen with a fenoterol solution was begun.

**The Effect of Fenoterol on the Production of Nitric Oxide**

**Fig. 3:**
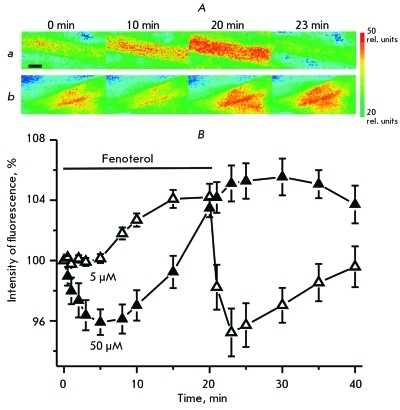
Effect of fenoterol on the synthesis of nitric oxide in cardiomyocytes of the isolated atrium. The changes in the fluorescence of the DAF-FM dye pre-loaded into cardiomyocytes of the isolated atrium, occurring as a response to the application of 5 ( *a* ) and 50 ( *b* ) µM fenoterol. The images of atrial cardiomyocyte bundles are presented (the intensity is shown with a pseudocolor). The scale is 10 µm. ( *B* ) The dynamics of the DAF-FM fluorescence (an indicator of nitric oxide production). Along Y-axis: fluorescence intensity (%) (100% corresponds to the value of fluorescence before application of fenoterol). Fenoterol concentrations of 5 ( *n*  = 6) and 50 ( *n*  = 6) µM are shown with open and filled triangles, respectively. The X-axis: time (min). The application of fenoterol is indicated with a line.

Under the action of fenoterol, the fluorescence of the DAF-FM marker (an indicator of NO production) in atrial cardiomyocytes reliably grew ( *Fig. 3* ). At low doses, fenoterol initiated a gradual increase in the NO production; and after 20 min of application of fenoterol, the intensity of the fluorescence grew to 104 ± 0.7% ( *p*  < 0.05, *n*  = 6). After the removal of fenoterol from the surrounding solution, the intensity of the fluorescence dropped to 95.0 ± 1.4% ( *p*  < 0.05, *n*  = 6) relative to the initial level, after which it returned to the baseline within 5 min. Under the action of a high dose of fenoterol, during the first 5 min of application, a decrease in the intensity of DAF-FM fluorescence was observed (by the fifth minute of application, the intensity was 95.9 ± 0.8% ( *p*  < 0.05, *n*  = 6)). After 8 min of application of fenoterol, the intensity of the fluorescence began to grow and reached 103.9 ± 0.6% ( *p*  < 0.05, *n*  = 6) within 20 min. After the atrial specimen was perfused with a fenoterol-free solution, the intensity of the fluorescence exceeded the control level for 10–15 min: its value was 104.3–106.7% ( *p*  < 0.05, *n*  = 6) relative to the baseline.

## DISCUSSION 

The slow development and stability of the effects produced by 1 and 5 µM of fenoterol indicate the activation of the intracellular signal systems with a long-term effect, which is caused by the stimulation of β _2_ -adrenoreceptors. In our opinion, the influence of fenoterol on the inotropic atrial function can be explained by the interaction of β _2_ -adrenoreceptors with several intracellular signaling cascades that regulate the contractility.

The key factor defining the contractility of the cardiac muscle is the concentration of calcium ions in cardiomyocytes during systole. The contractions of cardiomyocytes are initiated by calcium ions, which enter the cytosol in two ways. Depolarization of the membrane caused by the action potential leads to an opening of voltage-dependent calcium channels, through which calcium ions influx into the cytoplasm, where they bind to the ryanodine receptors (Ca-release channels) of the sarcoplasmic reticulum. As a result, calcium ions are released from the sarcoplasmic reticulum, and their concentration in the cytoplasm reaches a maximum. In our experiments, the application of fenoterol led to a significant increase in the intensity of Ca-signals ( *Fig. 2* ); this most likely occurs due to an increase in the amount of Ca ^2+^ ions entering through the channels of the plasma membrane. It is known that β _2_ -adrenoreceptors, as well as β _1_ -adrenoreceptors, bind to Gs proteins, which activate the adenylate cyclase that catalyzes the synthesis of cAMP, a secondary messenger. This process activates cAMP-dependent protein kinases, which phosphorylate the specific target proteins [37]. One of the main substrates for protein kinase A is the L-type calcium channel, which, in the phosphorylated state, has a higher conductance and a higher probability of opening up and is activated when the membrane potential values are more negative [9, 31].

The following interesting fact was denoted. At a concentration of 5 µM, fenoterol causes slow growth in the intensity of Ca-signals, whose amplitude remains high after the removal of fenoterol from the solution surrounding the specimen. Simultaneously, the application of 50 µM of fenoterol leads to an increase in the amplitude of Ca-signals ( *Fig. 2* ).

There are three possible explanations for this. First, it can be suggested that fenoterol begins to affect not only β _2_ -adrenoreceptors at a higher dose, but also β _1_ -adrenoreceptors, which activate an additional number of calcium channels under the action of protein kinase A. Thus, the constant characterizing the binding of fenoterol (stereoisomers *R,R* -1 and -2) to the β _1_ -adrenoreceptors of HEK cells is estimated to be approximately 15–20 µM [28]. In addition, the most effective concentration of fenoterol, i.e. the concentration that leads to a maximum increase in the contractility of human papillary muscle isolated specimens, was 100 µM; in this case, the effect of fenoterol depended on β _2_ - and β _1_ -adrenoreceptors [38]. However, this explanation contradicts our results, according to which the selective β _2_ -adrenoreceptor blocker (0.1 µM ICI-118.551) completely eliminates the positive inotropic effect produced by 50 µM fenoterol. The second possible explanation is the involvement of a large number of metabotropic β _2_ -adrenoreceptors that cause an increase in the production of cAMP and the activation of a large number of protein kinase A molecules, which in turn intensify the phosphorylation of Ca-channels, rendering them more active [1]. However, the *R,R* -stereoisomer of fenoterol at a concentration of around 0.5 µM activates almost all β _2_ -adrenoreceptors in rat ventricular cardiomyocytes[39]. According to the approximate estimations made, even at a concentration of 2 µM, the racemic fenoterol used occupies around 90% of the β _2_ -adrenoreceptors of bronchial epithelial cells [27]. Consequently, it is most likely that, under the action of 5 and 50 µM of fenoterol, an equal number of β _2_ -adrenoreceptors is activated. The third suggestion is based on the facts noted to date that, for transduction the signal inside a cell, β _2_ -adrenoreceptors can use different signal cascades and/or bind differently in terms of efficiency to effector molecules, depending on the dose of the agonist [22]. Thus, upon application of high doses of the agonist (100 µM isoproterenol), the isolated β _2_ -adrenoreceptors can exist in two different conformations [40]; in each conformation, the efficiency of the interaction of the activated receptor with G proteins and other proteins is different. It is interesting to note that as a response to the application of selectively high doses of the agonist, the β _2_ -adrenoreceptor begins to activate not only Gs proteins, but also tyrosine kinase Src, which is involved in the regulation of signal proteins (e.g., MAP-kinase) and phosphorylates the β _2_ -adrenoreceptor. The phosphorylated residues of β _2_ -adrenoreceptor tyrosine can play the role of a dockingsite for other signal molecules [22]. In addition, Src-kinase is capable of regulating (including the activation) L-type Ca-channel function by binding to the N-terminal region of the channel α1-subunit and phosphorylating it [41]. Most likely, the rapid increase in the amplitude of Ca-signals observed under the action of 50 µM of fenoterol occurs due to the activation of an additional pathway (e.g., involving Src-kinase), which facilitates the function of the L-type Ca-channels. In our opinion, the last suggestion is the most probable and should be studied experimentally in the first place.

In the study we performed, the increase in the production of nitric oxide in atrial cardiomyocytes occurring under the action of fenoterol was revealed for the first time ( *Fig. 3* ). The production of nitric oxide is an important factor that controls the contractility of cardiomyocytes. An intensification of the NO production may lead to a negative inotropic effect and/or interfere with increasing contractility of cardiomyocytes [42]. Such an effect by nitric oxide might be associated with the activation of cGMP-dependent protein kinases G, which phosphorylate troponin I, thereby decreasing the sensitivity of troponin C to calcium, and influence the Ca-channels, thereby decreasing the calcium conductance [43, 44]. Since the effect of nitric oxide is connected to the activation of the guanylate cyclase that is in the “on” state, it produces thousands of cGMP molecules; thus, even a slight increase in the synthesis of NO can have a significant influence on the cell functions [45].

Several hypotheses regarding the mechanisms of how β _2_ -adrenoreceptors are linked with the NO synthase responsible for the production of nitric oxide can be suggested. β _2_ -Adrenoreceptors have the ability to interact with the Gi protein. However, the activated receptor binds to the Gi protein less readily than it binds to the Gs protein [46]. It is assumed that for a longer period of time, the activated β _2_ -adrenoreceptors are in a state that is characterized by high affinity to the Gs protein, while it is in a state in which it is selectively bound to the Gi protein for a shorter period [47]. According to the other hypothesis, β _2_ -adrenoreceptors can interact with the Gi protein only after having been phosphorylated by protein kinase A or kinase of G protein coupled receptors [48]. The α-subunits of the Gi protein inhibit production of cAMP by adenylate cyclases, and the dimers of βγ-subunits affect some ionic channels and signal molecules; in particular, they activate the PI3K/protein kinase B pathway [1, 49]. One of the effectors in this pathway is NO synthase (neural and endothelial isoforms), whose activity intensifies after its phosphorylation by protein kinase B [50]. In rat endothelial cells, the application of fenoterol causes an increase in the PI3K/protein kinase B-dependent activity of the endothelial NO synthase [26]. However, in rat ventricular and cat atrial cardiomyocytes, fenoterol (0.1 µM) did not activate the pathway associated with the Gi protein and NO production, and its effect on contractility was exclusively associated with the Gs protein [15, 51].

The activation of the Gi-protein in HEK293 cells under the action of fenoterol has been recently found to be independent of ERK1/2 kinase (extracellular signal-regulating kinases) [29]. In rat cardiomyocytes, ERK1/2 is phosphorylated and activated in the NO-dependent pathway [52]. Most likely, β _2_ -adrenoreceptors are capable of activating NO synthase independently of the G-protein. The β-arrestin 2 protein, which interacts simultaneously with β _2_ -adrenoreceptors and NO synthase, may play the role of a factor that conjugates the activation of the receptor and the increase in NO production [43]. Src kinase stimulated by β _2_ -adrenoreceptors phosphorylates the Tyr83 residue of the endothelial NO synthase, thereby increasing the synthesis of NO [54]. In addition, the activation of NO production is facilitated by the complex of calcium ions with calmodulin [42]; that is why an increase in the production of nitric oxide is most likely partially caused by an increase in the concentration of calcium ions due to the high activity of L-type calcium channels.

It should be noted that in this work the production of nitric oxide started to increase almost immediately after the application of a low concentration of fenoterol, while at a high concentration, fenoterol caused a decrease, firstly, and then an increase in NO synthesis. Most likely, in the latter case, a dramatic increase in the production of cAMP firstly had a negative influence on the functioning of NO synthase (our unpublished data), and then the activating action of signals appeared from β _2_ -adrenoreceptors and an increased calcium level.

Upon consideration of the effects of the activation of β _2_ -adrenoreceptors, it should not be forgotten that β _2_ -adrenoreceptors can directly interact with the factor that regulates the Na/H exchange, the N-ethylmaleimide-sensitive factor (that controls the internalization of the receptors and the interaction with Gi-proteins), and the scaffold proteins that connect adrenoreceptors with protein kinase A, C, phosphatase 2A, and L-type calcium channels [20, 22–24]. As a result, the dimers of β _2_ -adrenoreceptors can accumulate around the macromolecular signal complex which provides a “coordinated” response by a cell to their activation [55, 56]. Moreover, β _2_ -adrenoreceptors, calcium channels, endothelial NO synthase, NADPH oxidase, and other regulatory molecules are colocalized in caveolae, small invaginations of the plasma membrane enriched in cholesterol and caveolin [55]. That is why the effects of β _2_ -adrenoreceptor activation *in vivo* can be associated with changes in the activity of a large number of regulatory molecules in limited regions of the cell.

β _2_ -Adrenoreceptors are known to exhibit a rapid loss of sensitivity to agonists, followed by a dramatic decrease in the number of receptors [27, 36]. Taking this into account, it can be suggested that fenoterol initiates processes that interfere with the desensitization of β _2_ -adrenoreceptors, which explains the stability of the effect of fenoterol on the amplitude of contractions and Ca-signals. Nitric oxide directly affects β _2_ -adrenoreceptors and the proteins involved in the desensitization of β _2_ -adrenoreceptors (G protein-coupled receptor kinases) via S-nitrosylation. As a result, it prevents a decline in the sensitivity of the receptors, which occurs under the action of the agonist, and a decrease in the amount of receptors [16, 53]. As a response to the activation of β _2_ -adrenoreceptors, dynamin, a small GTPase that participates in endocytosis, undergoes S-nitrosylation; this transformation boosts its ability to polymerize and form a contractive “collar” [57]. Thus, the prolonged effect of fenoterol might be explained by the action of nitric oxide on receptors, G protein-coupled receptors, and dynamin. On the one hand, the increase in NO production that occurs under the action of fenoterol facilitates the closing of caveolae (i.e. the narrowing of the pore that connects an extracellular medium with the cavity of caveolae), in which the agonist molecules are captured; and on the other hand, it provides long-term activity of β _2_ -adrenoreceptors. In this case, even the removal of fenoterol from an extracellular medium does not cause a rapid decline in the effects.

## CONCLUSIONS 

Relying on the data obtained in this work, the following can be suggested. The activation of β _2_ -adrenoreceptors with low doses of the agonist simultaneously initiates signal cascades, which have differently directed effects on atrial contractility. That is why the positive inotropic effect produced by the agonist does not occur at the beginning. However, the production of nitric oxide declines, while the amplitude of Ca-signals remains high; this leads to an increase in contractility. In the case of activation of β _2_ -adrenoreceptors with high doses of the agonist, the pathway associated with a dramatic increase in the amplitude of Ca-signals is activated first, while the increase in the production of nitric oxide is “delayed”; that is why a significant, positive inotropic effect of the agonist is observed. The hypothetic two-component differently directed mechanism which underlies the changes in the atrial contractility occurring upon activation of β-adrenoreceptors by fenoterol (racemic) requires a more detailed experimental study. The pharmacological approach involving the application of nitric oxide synthesis blockers, the adenylate cyclase system, L-type calcium channels, ryanodine receptors, and probably endocytosis inhibitors would allow to shed more light on the pathways of the effects produced by the β _2_ -adrenoreceptors of atrial cardiomyocytes, thereby providing an answer to the questions raised in this work.
